# Differentially expressed genes and interacting pathways in bladder cancer revealed by bioinformatic analysis

**DOI:** 10.3892/mmr.2014.2396

**Published:** 2014-07-18

**Authors:** YINZHOU SHEN, XUELEI WANG, YONGCHAO JIN, JIASUN LU, GUANGMING QIU, XIAOFEI WEN

**Affiliations:** Department of Urology, Shanghai East Hospital, Tongji University School of Medicine, Shanghai 200120, P.R. China

**Keywords:** bladder cancer, differentially expressed genes, interacting pathways, enrichment analysis, immune and inflammation-related pathways

## Abstract

The goal of this study was to identify cancer-associated differentially expressed genes (DEGs), analyze their biological functions and investigate the mechanism(s) of cancer occurrence and development, which may provide a theoretical foundation for bladder cancer (BCa) therapy. We downloaded the mRNA expression profiling dataset GSE13507 from the Gene Expression Omnibus database; the dataset includes 165 BCa and 68 control samples. T-tests were used to identify DEGs. To further study the biological functions of the identified DEGs, we performed a Kyoto Encyclopedia of Genes and Genomes (KEGG) pathway enrichment analysis. Next, we built a network of potentially interacting pathways to study the synergistic relationships among DEGs. A total of 12,105 genes were identified as DEGs, of which 5,239 were upregulated and 6,866 were downregulated in BCa. The DEGs encoding activator protein 1 (AP-1), nuclear factor of activated T-cells (NFAT) proteins, nuclear factor κ-light-chain-enhancer of activated B cells (NF-κB) and interleukin (IL)-10 were revealed to participate in the significantly enriched immune pathways that were downregulated in BCa. KEGG enrichment analysis revealed 7 significantly upregulated and 47 significantly downregulated pathways enriched among the DEGs. We found a crosstalk interaction among a total of 44 pathways in the network of BCa-affected pathways. In conclusion, our results show that BCa involves dysfunctions in multiple systems. Our study is expected to pave ways for immune and inflammatory research and provide molecular insights for cancer therapy.

## Introduction

Bladder cancer (BCa) is a heterogeneous disease with a variable disease history. At present, BCa is the ninth most common tumor worldwide (1), ranking fourth among men and ninth among women (2). In the United States, ~10,400 new BCa cases emerge and 36,500 BCa-related deaths occur every year (3). The diseases of urothelial carcinoma can be classified depending on their depth of invasion as: pTa (papillary), pT1 (lamina propria invasion), pT2 (muscle invasion), pT3 (invasion to peri-vesical fat) and pT4 (locally advanced).

BCa has a multifactorial etiology. Numerous studies have identified risk factors for the development of BCa and the affected cellular processes, including low-penetrance gene polymorphisms, smoking (4), occupational exposure to aromatic amines and schistosomiasis infection (5,6). In addition, the fact that men are 3–4 times more frequently affected than women indicates hormone regulation as an additional factor (7,8).

The detection of BCa remains difficult due to the lack of specific tumor markers and the emergence of new symptoms. There is no standard therapy for patients with BCa; treatment options include surgery, chemo-, biological and radiation therapy. In addition, these methods show currently limited effectiveness, which is further reduced by the high recurrence of BCa and the high mortality deriving from increased rates of progression of the disease (9). There is thus an urgent need to develop more specific and efficient diagnostic tools and therapeutic approaches to better understand and treat the onset and course of BCa.

Earlier studies have suggested that different mechanisms have evolved to respond to specific phenotype alterations in the molecular and cellular pathways involved in BCa. A number of genetic variations in major carcinogenesis-related pathways can induce BCa. For example, in healthy cells, the cell cycle is controlled by tightly linking the p53 and retinoblastoma (RB) pathways that influence regulation of apoptosis, signal transduction and gene expression. During cancer progression and metastasis, the mutated p53 protein exerts profound changes in regulatory networks involved in cell adhesion and angiogenesis, and becomes resistant to degradation in BCa of pT2–pT4 (10). However, the specific mechanisms underlying the progression of BCa are still unclear, and therefore, identifying genetic markers of the disease will allow the development of effective treatment and detection methods for early-stage bladder tumors.

In the present study, we identified genes the expression of which is significantly changed between healthy and cancer cells and discuss the biological functions of these genes and the related pathways. In addition, we built a network of BCa-affected pathways in order to explore the mechanisms underlying the pathogenesis, occurrence and development of BCa. Our results may provide valuable molecular insights for BCa treatment.

## Materials and methods

### mRNA expression profiling data

The mRNA expression profiling dataset for BCa was downloaded from the Gene Expression Omnibus (GEO) database (accession no. GSE13507) at the National Center of Biotechnology Information. The dataset comprises a total of 256 samples, including 10 healthy bladder mucosae, 58 normal-looking bladder mucosae surrounding cancer, 165 primary bladder cancer and 23 recurrent non-muscle invasive tumor tissues. Gene expression was previously studied in these samples on an Illumina Human-6 v2.0 expression beadchip platform (11,12).

### Data processing

The raw expression data were downloaded as a Series Matrix file. This expression matrix contained 43,148 probes. Among these, a total of 24,995 probes uniquely mapped to 24,340 genes. For different probes mapping to the same gene, we the mean probe value was calculated for the gene expression value. An expression matrix (24,340*234) was thus obtained, with each row representing gene expression value, and each column representing the sample. From the 256 samples, we selected 233, corresponding to 165 primary BCa and 68 control samples (healthy bladder and normal-looking bladder mucosae surrounding the cancer).

### Identification of differentially expressed genes (DEGs)

A statistical t-test was used to identify DEGs between BCa and control samples and to calculate the associated p-values. Multiple testing correction on these p-values was performed with the Benjamini and Hochberg method (13), and provided false discovery rates (FDRs). DEGs with an FDR<0.05 were selected for further analyses.

### Kyoto Encyclopedia of Genes and Genomes (KEGG) pathway enrichment analysis

KEGG is a knowledge database for the systematic analysis of gene functions, linking genomic information with high-order functional information. To identify significantly enriched KEGG pathways among the DEGs, we first downloaded from the database 232 KEGG pathways on April 26th, 2011 (14). We next used a hypergeometric distribution model to test the statistical significance of the hypothesis that all pathways are co-disrupted in the same samples. The resulting P-value was calculated as follows:

$$\text{p}=1-\sum_{0}^{k-1}\frac{C_{k}^{m}C_{n-k}^{N-m}}{C_{\text{n}}^{N}}$$

In this formula, N is the total number of gene samples involved in KEGG pathways; n is the number of differentially expressed gene samples; m is the number of gene samples in one pathway; and k is the frequency of overlapping genes in all pathways. FDR<0.01 was set as the threshold.

### Identification of potentially interacting pathways

In order to explore potential synergistic relationships between BCa-affected pathways, we constructed the pathway network in which nodes represent pathways and edges represent nonrandom synergistic relationships (crosstalk interactions). The network was built using Cytoscape (15). The crosstalk among pathways was then investigated by calculating the Jaccard index (Ji), similarly to a previous study (16). The Ji is defined as follows:

$$\text{Jaccard}(\text{path A},\text{path B})=\frac{\#\text{intersection }(\text{path A},\text{path B})}{\#\text{union }(\text{path A},\text{path B})}$$

In this formula, #intersection (path A, path B) represents the overlapping number of genes between pathway A and B, and #union (path A, path B) represents the sum of genes in pathway A and B. All pathway pairs were grouped together to construct the network. Pathways that showed Ji≥0.01 and shared at least one DEG were selected.

## Results

### Identification of DEGs

Based on t-tests and a threshold FDR from multiple testing correction <0.05, we identified 12,105 DEGs, 5,239 upregulated and 6,866 downregulated.

### KEGG pathway enrichment analysis

In order to gain further insights into the biological functions of the identified DEGs, we used a hypergeometric distribution test to identify the significantly enriched pathways related to BCa. Using a threshold FDR<0.05, we found that upregulated DGEs are significantly enriched for 7 pathways (Table I), while downregulated DGEs are significantly enriched for 47 pathways (Table II).

### Identification of interacting pathways of DGEs

**Figure 1:**
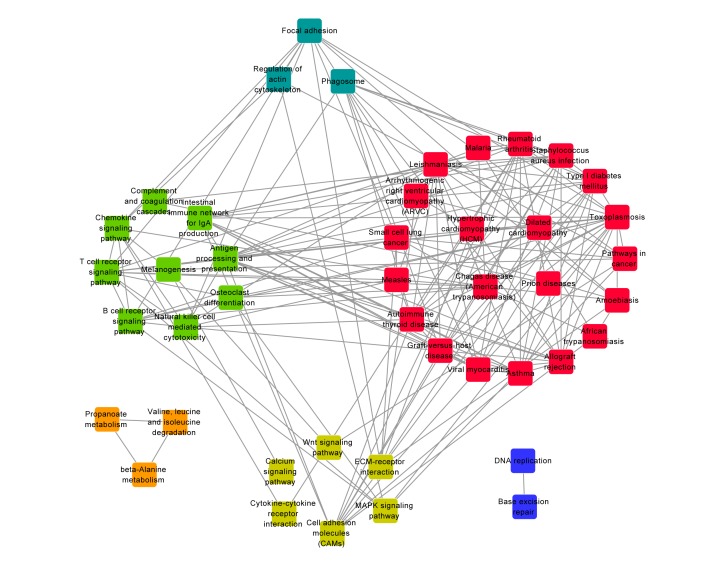
The network of the 44 PCa-affected pathways and interactions between these. Bottle green boxes, cellular process pathways; yellow, environmental information processing pathways; red, human disease-related pathways; green, organismal systems pathways; orange, metabolic pathways; and blue, information processing pathways.

In order to systematically analyze the DEG functions, we used a Jaccard model to construct and identify the interactions between the BCa-affected pathways. We selected the pathways that showed Ji ≥ 0.01 and shared at least one DGE, and thus identified, from 172 pathway pairs, a total of 44 pathways that were considered good candidates for a crosstalk interaction (Fig. 1). Among these, a number of pathways relate to functional disorders and could thus be relevant to BCa.

### Exploring the mechanisms underlying BCa through KEGG pathways

**Figure 2:**
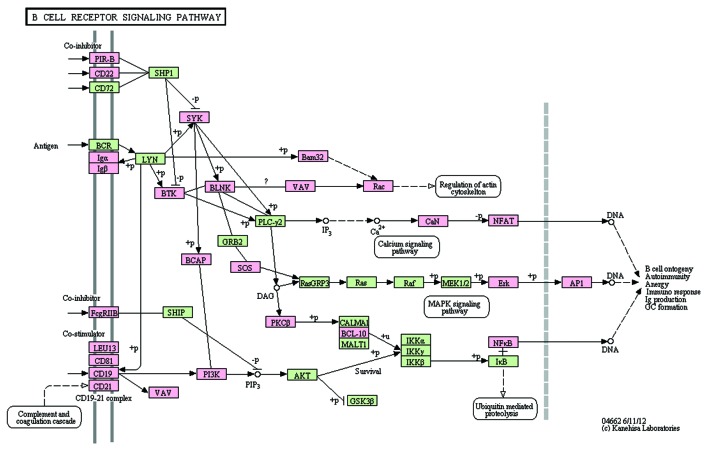
Differentially expressed genes (DEGs) in the B-cell receptor signaling pathway. Pink boxes, DEGs; green, non differentially expressed genes. Source: Kyoto Encyclopedia of Genes and Genomes.

**Figure 3:**
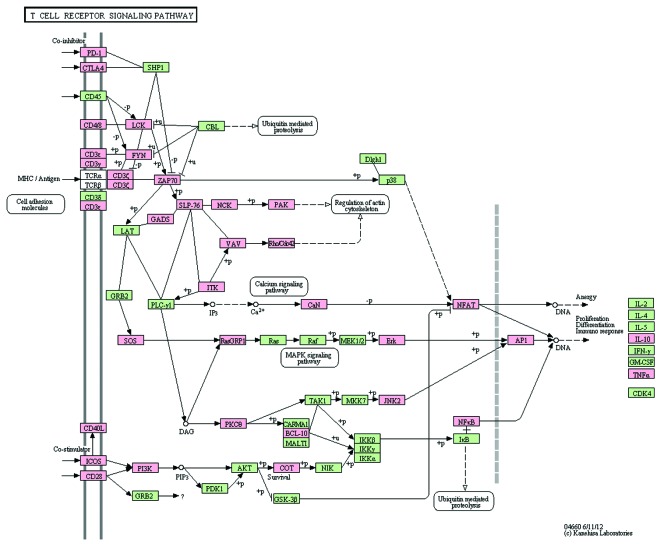
Differentially expressed genes (DEGs) in the T-cell receptor signaling pathway. Pink boxes, DEGs; green, non-differentially expressed genes. Source: Kyoto Encyclopedia of Genes and Genomes.

To investigate the mechanism underlying the BCa disorder, we focused on the immune- and inflammation-related pathways identified within the network of interacting pathways. These included chemokine signaling, complement and coagulation cascades, antigen processing and presentation pathways, natural killer cell-mediated cytotoxicity, T- and B-cell receptor signaling pathways, and the intestinal immune network for IgA production. Furthermore, in order to assess the role of immune responses in the development of cancer, we focused on the B- and T-cell receptor signaling pathways. We found that the B-cell receptor signaling pathway is downregulated in BCa, involving 36 DGEs (Fig. 2). The T-cell receptor signaling pathway is also downregulated, and contains 46 DGEs (Fig. 3).

## Discussion

BCa, which is pathologically diagnosed as transitional or urothelial carcinoma, is the only known solid tumor besides melanoma. Detection and treatment of BCa remain a challenge. Therefore, there is an urgent need to explore the mechanisms involved in BCa and develop an effective preventive strategy for this disorder. In the present study, we first identified DEGs by comparing mRNA expression profiling data (downlooaded from the GEO database) between cancer and control samples and then applied a hypergeometric distribution test to identify the significantly enriched pathways these DEGs are involved in. Furthermore, we constructed a network of the relevant pathways and assessed their potential interactions.

The KEGG pathway enrichment analysis showed that the upregulated DEGs are related to 7 pathways. Among these, the most significantly enriched is the cell-cycle regulation pathway, which is a signal transduction cascade triggered in response to the detection of DNA damage (17), and which has been demonstrated to relate to the development of BCa. It has been demonstrated that cell-cycle regulatory pathways function as checkpoints that control the order and timing of cell- cycle transitions to ensure the fidelity of critical events, such as DNA replication and chromosome segregation (18). Cells may be arrested at any phase of the cycle, temporarily halting the cell cycle and allowing DNA repair to be completed. Loss and/or perturbation of cell-cycle control checkpoints results in genomic instability and is a hallmark of cancer, as evidenced by the common association of polymorphisms/inactivation of cell-cycle control genes such as p53, p16 and Rb1, with various types of cancer (19,20). Other pathways that were found as significantly enriched among upregulated DEGs were: those involved in cell proliferation, which is controlled by proteins that maintain the synchrony of cell growth, DNA synthesis, mitosis and cell division (21,22); base excision repair, which is related to DNA repair mechanisms; and lysine degradation, which is involved in a wide range of metabolic disorders.

The downregulated DEGs were found as involved in 47 significantly enriched pathways, mainly including cell transfer-related pathways, such as: cell adhesion molecules and focal adhesion; cell growth-related pathways, such as WNT, transforming growth factor-β (TGF-β), mitogen-activated protein kinase (MAPK) and calcium signaling pathways; and cancer-related immune and inflammation pathways, such as the T-/B- cell receptor and the chemokine signaling pathways. Epidemiological studies have estimated that underlying infections and inflammatory responses are linked to 15–20% of cancer-related deaths worldwide (23). The intrinsic immune pathway is activated by genetic events that cause neoplasia. Cancer cells produce inflammatory mediators, thereby generating an inflammatory microenvironment in the tumor.

To explore the potentially synergistic interactions among the BCa-affected pathways, we constructed a network based on significantly enriched pathways and Ji calculations. The crosstalk interactions revealed in this network indicated that cancer incidence relates to alterations in multiple processes, including environmental and genetic information processing, metabolism, organismal systems, and certain human diseases. These processes may synergistically contribute to the regulation and progression of BCa.

To further reveal the mechanisms underlying the BCa disorder, we focused on immune- and inflammation-related pathways. Our analysis indicated that natural killer cell-mediated cytotoxicity and the B- and T-cell receptor signaling pathways are associated with the formation of bladder tumors and may participate in the development of BCa. Numerous studies have demonstrated that structures on the transformed cell (either expressed as a result of the transformation process itself or induced by the ongoing but limited inflammatory response) are recognized by infiltrating lymphocytes such as natural killer T-, natural killer or γδ T-cells, which are then stimulated to produce interferon-γ (IFN-γ) (24,26). The IFN-γ that is initially produced may induce a limited amount of tumor death through antiproliferative (27) and apoptotic mechanisms. A previous study demonstrated that the bacillus Calmette-Guerin (BCG) vaccine plays an important role in the maturation of dendritic cells (DCs) by signaling through different Toll-like receptors, as indicated by upregulation of the DC maturation marker CD83 (28) and secretion of inflammatory cytokines such as interleukin (IL)-12, IFN-γ and tumor necrosis factor-α, suggesting a predominant involvement of the T-helper 1 lymphocyte subpopulation in inflammation. Tumor-specific CD4 and CD8 T-cells home to the tumor along a chemokine gradient where they recognize and destroy tumor cells expressing distinct tumor antigens. Furthermore, natural killer, lymphocyte-activated and BCG-activated killer cells and macrophages can kill bladder tumor cells *in vitro* (29).

Activator protein 1 (AP-1), involved in cellular proliferation, transformation and death (30), is a dimeric complex that contains members of the JUN, FOS, ATF and MAF protein families. AP-1 proteins are primarily considered to be oncogenic, but recent studies have shown that they can also have a tumor-suppressor activity (31). It was further demonstrated that members of the AP-1 complex and ATF2 synergistically contribute to tumorigenesis (32). Nuclear factor of activated T-cells (NFAT) is a protein family first identified more than two decades ago as a major stimulation-responsive DNA-binding factor and transcriptional regulator family in T cells. It is now established that NFAT proteins play important roles in additional types of immune cells and regulate numerous developmental processes in vertebrates. Dysregulation of these processes can lead to malignant growth and cancer (33). The nuclear factor κ-light-chain-enhancer of activated B cells (NF-κB) is activated by a variety of cancer-promoting agents. The reciprocal activation between NF-κB and inflammatory cytokines renders NF-κB an important factor for inflammation-associated cancer development. Both the constitutive and anticancer therapeutic agent-induced NF-κB activation blocks the anticancer activities of the therapeutic agents. Elucidating the roles of NF-κB in cancer facilitates the development of approaches for cancer prevention and therapy (34). A recent study showed that IL-20 promotes the migration of BCa cells by inducing ERK-mediated matrix metalloproteinase-9 expression, leading to the activation of NF-κB through the upregulation of p21 (WAF1) (35). IL-10 is the most important cytokine with anti-inflammatory properties besides TGF-β and IL-35. It is produced by activated immune cells and regulates the functions of numerous and distinct types of immune cells. In addition, IL-10 plays an important role in the biology of B and T cells. The physiological relevance of this cytokine lies in the prevention and limitation of specific and unspecific immune reactions and, consequently, of tissue damage (36). One study revealed the inhibitory role of IL-10 in BCG-induced macrophage cytotoxicity, suggesting that blocking IL-10 may potentially enhance the effect of BCG in the treatment of BCa patients (37).

Overall, the present study presented a comprehensive bioinformatic analysis of genes and pathways that may be involved in the progression of BCa. Our results on immune- and inflammation-related pathways affected by BCa may prove helpful in the development of a new strategy to treat BCa in combination with medical therapy. Considering the increasing public availability of genomic data, we argue that our approach will constitute an attractive strategy for identifying disease-related pathways and interactions in numerous future studies. However, further experimental studies are needed to reveal the molecular mechanisms underlying BCa-associated processes.

## Figures and Tables

**Table I:** Significantly enriched pathways among the upregulated differentially expressed genes (DEGs).

KEGG id.	Pathway name	Genes	DEGs	P-value	BH^a^
hsa04110	Cell cycle	128	62	5.11E-08	1.19E-05
hsa05322	Systemic lupus erythematosus	138	57	7.80E-05	9.05E-03
hsa03008	Ribosome biogenesis in eukaryotes	84	38	1.30E-04	1.01E-02
hsa03030	DNA replication	36	20	1.83E-04	1.06E-02
hsa03410	Base excision repair	34	19	2.39E-04	1.11E-02
hsa00310	Lysine degradation	44	22	6.41E-04	2.48E-02
hsa03013	RNA transport	153	58	9.74E-04	3.23E-02

a q-value from Benjamini and Hochberg multiple testing connection.

KEGG, Kyoto Encyclopedia of Genes and Genomes.

**Table II:** Significantly enriched pathways among the downregulated differentially expressed genes (DEGs).

KEGG id.	Pathway name	Genes	DEGs	P-value	BH^a^
hsa04640	Hematopoietic cell lineage	88	49	2.93E-08	6.79E-06
hsa05330	Allograft rejection	39	27	8.31E-08	9.64E-06
hsa05416	Viral myocarditis	72	41	1.73E-07	1.33E-05
hsa05150	*Staphylococcus aureus* infection	56	34	2.39E-07	1.39E-05
hsa05332	Graft-versus-host disease	43	28	3.51E-07	1.63E-05
hsa04514	Cell adhesion molecules	136	65	4.32E-07	1.67E-05
hsa04510	Focal adhesion	200	87	9.64E-07	2.83E-05
hsa04612	Antigen processing and presentation	78	42	9.76E-07	2.83E-05
hsa05323	Rheumatoid arthritis	92	47	1.66E-06	4.29E-05
hsa04940	Type I diabetes mellitus	45	27	5.81E-06	1.35E-04
hsa05145	Toxoplasmosis	133	60	1.22E-05	2.58E-04
hsa04672	Intestinal immune network for IgA production	49	28	1.44E-05	2.79E-04
hsa05140	Leishmaniasis	73	36	7.18E-05	1.28E-03
hsa05310	Asthma	31	19	9.62E-05	1.59E-03
hsa05144	Malaria	51	27	1.25E-04	1.93E-03
hsa04662	B-cell receptor signaling pathway	75	36	1.46E-04	2.11E-03
hsa00280	Valine, leucine and isoleucine degradation	44	24	1.61E-04	2.19E-03
hsa05146	Amoebiasis	106	47	1.76E-04	2.26E-03
hsa04145	Phagosome	156	64	2.16E-04	2.64E-03
hsa03010	Ribosome	92	41	3.92E-04	4.29E-03
hsa04020	Calcium signaling pathway	177	70	4.07E-04	4.29E-03
hsa04062	Chemokine signaling pathway	189	74	4.01E-04	4.29E-03
hsa05320	Autoimmune thyroid disease	54	27	4.26E-04	4.30E-03
hsa05142	Chagas disease (American trypanosomiasis)	104	45	4.66E-04	4.51E-03
hsa04512	ECM-receptor interaction	85	38	5.87E-04	5.45E-03
hsa04660	T-cell receptor signaling pathway	108	46	6.17E-04	5.51E-03
hsa00640	Propanoate metabolism	32	18	6.49E-04	5.58E-03
hsa04610	Complement and coagulation cascades	69	32	7.21E-04	5.97E-03
hsa05020	Prion diseases	35	19	8.35E-04	6.46E-03
hsa05340	Primary immunodeficiency	35	19	8.35E-04	6.46E-03
hsa05200	Pathways in cancer	327	116	1.13E-03	8.47E-03
hsa05412	Arrhythmogenic right ventricular cardiomyopathy	74	33	1.39E-03	1.01E-02
hsa04060	Cytokine-cytokine receptor interaction	275	99	1.50E-03	1.05E-02
hsa04310	WNT signaling pathway	151	59	1.59E-03	1.08E-02
hsa04810	Regulation of actin cytoskeleton	214	79	1.99E-03	1.32E-02
hsa05410	Hypertrophic cardiomyopathy	87	37	2.10E-03	1.35E-02
hsa04350	TGF-β signaling pathway	85	36	2.61E-03	1.59E-02
hsa05143	African trypanosomiasis	35	18	2.56E-03	1.59E-02
hsa04380	Osteoclast differentiation	128	50	3.52E-03	2.09E-02
hsa05414	Dilated cardiomyopathy	90	37	4.22E-03	2.45E-02
hsa04010	MAPK signaling pathway	272	95	5.19E-03	2.86E-02
hsa05222	Small cell lung cancer	85	35	5.15E-03	2.86E-02
hsa04650	Natural killer cell mediated cytotoxicity	140	53	5.78E-03	3.12E-02
hsa05162	Measles	134	51	5.94E-03	3.13E-02
hsa04916	Melanogenesis	101	40	6.49E-03	3.35E-02
hsa00410	β-alanine metabolism	27	14	6.91E-03	3.48E-02
hsa00604	Glycosphingolipid biosynthesis-ganglio series	15	9	8.93E-03	4.41E-02

a q-value from Benjamini and Hochberg multiple testing connection.

KEGG, Kyoto Encyclopedia of Genes and Genomes; ECM, extracellular matrix; TGF-β, transforming growth factor-β; MAPK, mitogen-activated protein kinase.
